# Refolding and purification of recombinant L-asparaginase from inclusion bodies of *E. coli* into active tetrameric protein

**DOI:** 10.3389/fmicb.2014.00486

**Published:** 2014-09-15

**Authors:** Arun K. Upadhyay, Anupam Singh, K. J. Mukherjee, Amulya K. Panda

**Affiliations:** ^1^Product Development Cell, National Institute of ImmunologyNew Delhi, India; ^2^School for Biotechnology, Jawaharlal Nehru UniversityNew Delhi, India

**Keywords:** L-asparaginase-II, *Escherichia coli*, IBs, mild solubilization, refolding

## Abstract

A tetrameric protein of therapeutic importance, *Escherichia coli* L-asparaginase-II was expressed in *Escherichia coli* as inclusion bodies (IBs). Asparaginase IBs were solubilized using low concentration of urea and refolded into active tetrameric protein using pulsatile dilution method. Refolded asparaginase was purified in two steps by ion-exchange and gel filtration chromatographic techniques. The recovery of bioactive asparaginase from IBs was around 50%. The melting temperature (Tm) of the purified asparaginase was found to be 64°C. The specific activity of refolded, purified asparaginase was found to be comparable to the commercial asparaginase (190 IU/mg). Enzymatic activity of the refolded asparaginase was high even at four molar urea solutions, where the IB aggregates are completely solubilized. From the comparison of chemical denaturation data and activity at different concentrations of guanidine hydrochloride, it was observed that dissociation of monomeric units precedes the complete loss of helical secondary structures. Protection of the existing native-like protein structure during solubilization of IB aggregates with 4 M urea improved the propensity of monomer units to form oligomeric structure. Our mild solubilization technique retaining native-like structures, improved recovery of asparaginase in bioactive tetrameric form.

## Introduction

Most of the times, expression of recombinant proteins in *Escherichia coli* leads to the formation of insoluble aggregates known as IBs (Hartley and Kane, 1988; Fahnert et al., 2004). Recovery of active protein from IB aggregates remains to be a cumbersome task and requires standardization of solubilization and refolding methods (De Bernardez et al., 1999; Burgess, 2009). The major hurdle associated with purification of proteins from IBs is the sub-optimal refolding of recombinant proteins into native conformation (Rudolph and Lilie, 1996; Panda, 2003; Vallejo and Rinas, 2004). Poor refolding is often associated to high concentrations of urea or guanidine hydrochloride (GdmCl) used to solubilize the IB proteins. At higher concentrations, chaotropes such as urea and GdmCl completely denature the proteins and increases its propensity to aggregate during refolding resulting in low recovery of bioactive protein from IBs. IB proteins are reported to have structure and functional activities (Umetsu et al., 2004; Ventura and Villaverde, 2006; Peternel and Komel, 2011). These active IBs can be isolated from bacterial cells using different methods like homogenization, enzymatic lysis, and sonication where homogenization was observed to be most appropriate (Peternel and Komel, 2010). It will be ideal to protect these secondary protein structures during IB solubilization process. Mild solubilization of IB aggregates protects the existing native-like protein structure of IB proteins and helps in its improved recovery into bioactive form Singh and Panda (2005). In a few cases, solubilization with mild denaturing conditions has been proved to be more efficient for the recovery of bioactive protein from the IBs (Panda, 2003; Singh et al., 2012; Upadhyay et al., 2012).

Refolding yield of active oligomeric proteins from IBs is even lower (Scrofani et al., 2000; Karumuri et al., 2007; Garrido et al., 2011). Formation of active monomer and its association is a prerequisite for refolding into fully active oligomeric proteins. Often it is hindered due to complete unfolding of proteins in IBs into random coil structure while using high concentration of chaotropes. Solubilized protein molecules have propensity to form intermolecular aggregates leading to massive aggregation during refolding. It is thus essential to protect the secondary helical structure of IB proteins so that it reduces intermolecular aggregation between monomers. It can be achieved by adopting mild solubilization process for solubilization of IBs. Chemical denaturation studies provide information about the solubility profile of IB aggregates. Based on this information, IBs can be solubilized at low denaturation concentration while protecting the existing native-like secondary protein structure. Refolding of protein from monomers having secondary structural element will promote monomer association leading to the formation of active oligomeric protein and thus will improve the overall recovery of bioactive protein from IBs. Even though mild solubilization processes have been used to recover bioactive protein from IBs, there is very little information available on the refolding of oligomeric proteins into bioactive form.

Bacterial asparaginases from *Escherichia coli* and *Erwinia chrysanthemi* have been extensively used as drugs for the treatment of acute lymphoblastic leukemia (Muller and Boos, 1998; Graham, 2003; Verma et al., 2007). L-asparaginases (EC 3.5.1.1) catalyze the hydrolysis of L-asparagine to L-aspartic acid and ammonia. All asparaginases consist of four identical subunits A, B, C, and D and exist in homo-tetrameric form (Kozak et al., 2002) having masses in the range of 140–150 kDa (Aung et al., 2000). One subunit consists of two α/β domains that are connected by linking sequence. Interaction between N and C-terminal domain of adjacent monomers forms each active site. Therefore, the asparaginase tetramer can be treated as dimer of dimers because active site is either created by subunits A and C or B and D. The active form of the enzyme is a tetramer, and the dimers lack enzyme activity (Swain et al., 1993). L-asparaginase contains one tryptophan molecule at 66 positions in each monomer. It consists of four active sites formed at the interfaces of N and C- terminal domains of two interacting monomers (Swain et al., 1993). L-asparaginase has been reported to be produced using recombinant *E. coli* (Khushoo et al., 2004; Oza et al., 2011), *P. pastoris* (Ferrara et al., 2010) and from other microorganisms (Mahajan et al., 2012). Most of the times, the enzymes is produced in soluble form and purified with 40–60% recovery. There are no reports till date on solubilization and refolding of L-asparaginase from IBs into bioactive tetrameric form.

In this work, *E. coli* L-asparaginase II was expressed in *E. coli* cells as IBs and used as a model system to refold into bioactive tetrameric form. Attempts were made to solubilize the IBs of asparaginase in mild denaturing conditions. Solubilized proteins were then refolded and purified into active oligomeric form. Role of structural integrity between monomers and their importance in regulation of enzyme activity and stability was monitored by chemical, pH, temperature and organic solvent based denaturation methods. The results are of indication that mild solubilization followed by pulsatile refolding leads to improved recovery of bioactive multimeric L-asparaginase from IBs of *E. coli.*

## Materials and methods

### Chemicals

Culture media ingredients, tryptone, and yeast extract were from Difco Laboratories, India. Tris buffer, glycine, IPTG, sodium dodecyl sulfate, PMSF, and deoxycholic acid were from Amresco, USA. Ammonium persulfate, acrylamide, bis-acrylamide, *E. coli* asparaginase, L-arginine and Urea were from Sigma Chemicals, USA. DEAE-sepaharose and S-200 gel filtration matrix was from GE Healthcare, Sweden. TEMED, EDTA, bromophenol blue from Biorad, USA. Coomassie brilliant blue R-250 and ampicillin from USB Corporation, Cleveland, Ohio. Glucose, NaCl, Nessler's reagent, and other chemicals were from Qualigen, India.

### *Escherichia coli* strains and cloning of L-asparaginase

*Escherichia coli* BL21 (DE3) strain was from Novagen, USA. *E. coli* DH5α strain was obtained from Amersham Bioscience, USA. Plasmid pET14b was from Novagen, USA. L-asparaginase II (ansB) gene was amplified from the genomic DNA of *E. coli* K-12 strain (JM109) using primers (forward) 5′GTGCAGCACATATGTTACCCAATA TCACCA 3′and (reverse) 5′ GGCGGGATCCTTAGTACTGATTGAAGA 3′. NdeI and BamHI restriction sites were incorporated in the primers to facilitate cloning of the structural asparaginase gene (without its native signal sequence) in the *E. coli* expression vector pET14b. *E. coli* BL21 (DE3) cells were transformed with recombinant pET14b plasmid vector and used for expression of L-asparaginase II.

### Expression and isolation of asparaginase IBs

Transformed *E. coli* BL21 (DE3) cells were grown in modified LB media (1% tryptone, 0.5% yeast extract, 0.5% glucose and 1% NaCl) with 100 μg/ml ampicillin at 37°C in incubator shaker at 200 rpm. Culture at an OD_600_ of 0.6 was induced with 1 mM IPTG and was further grown for 3.5 h. Cells (1 L culture) were pelleted at 6000 rpm (rotor SLA-3000, Sorvall evolution RC) for 10 min at 4°C. Supernatant was discarded and culture pellet was resuspended in 20 ml buffer I (50 mM Tris-HCl, 1 mM EDTA, 1 mM PMSF, pH 8.5) containing egg white lysozyme (1 mg/ml) and incubated at room temperature for 2 h. Re-suspended cells were sonicated for 10 cycle (1 min cycle with 1 min gap) using Branson sonifier 450, Germany (probe diameter 13 mm, output voltage 60 and 50% duty cycle). Sonicated cells were centrifuged at 12,000 rpm (SA-300 rotor, Sorvall evolution RC) for 20 min at 4°C and the pellet containing recombinant asparaginase as IBs was separated. These pellets were resuspended in 20 ml buffer I containing 0.1% (w/v) deoxycholic acid, sonicated and centrifuged as described earlier (Patra et al., 2000). Pellet was further washed two times with buffer I and once with de-ionized water. Washed pellet (purified IBs) was resuspended in 1 ml 50 mM Tris-HCl buffer, pH 8.5 and used for solubilization and refolding.

### Solubilization and refolding of asparaginase from IBs

Isolated asparaginase IBs (1 ml) were solubilized in 9 ml buffer II (4 M urea, 50 mM Tris-HCl, 1 mM PMSF, 20 mM β-mercaptoethanol, 10 mM NaCl, pH 8.5) and kept for one h at room temperature. Solubilized asparaginase was centrifuged at 15,000 rpm for 30 min and supernatant was filtered through 0.2 μm filter (Millipore, USA). Solubilized protein was refolded into 90 ml buffer III (0.5 M urea, 50 mM Tris-HCl, 0.1 M arginine, 10 mM NaCl) by pulsatile dilution method at flow rate of 0.1 ml/min at 4°C. Refolded sample was centrifuged at 15,000 rpm (rotor SA-300, Sorvall evolution RC) for 30 min at 4°C. Supernatant was collected and dialyzed against buffer (50 mM Tris-HCl, 0.5 M urea, pH 8.5) using dialysis tubing (10 kDa, molecular weight cut-off) for 4 h at 4°C. Buffer was exchanged three times with 50 mM Tris-HCl, pH 8.5, at 4 h interval. Dialyzed protein was pooled and used for purification.

### Purification of refolded recombinant L-asparaginase

Refolded L-asparaginase was purified using DEAE-sepharose anion exchange matrix packed in XK 16 column (GE Healthcare, Sweden). Refolded asparaginase was loaded on to the column at flow rate of 1 ml/min and column was washed with 3 bed volumes of 50 mM Tris-HCl buffer at pH 8.5. Recombinant asparaginase was eluted from column using 0 to 0.5 M continuous gradient of NaCl. Fractions (2 ml each) were collected and analyzed by 12% SDS polyacrylamide gel electrophoresis. Ion exchange fractions containing asparaginase were pooled and concentrated by ultra-filtration (10 kDa molecular weight cut-off) to the final volume 5 ml. Concentrated asparaginase was loaded on S-200 Sephacryl column (XK16/60, GE Healthcare, Sweden) pre-equilibrated with 50 mM Tris-HCl, 10 mM NaCl, pH 7.5 buffer at flow rate 0.5 ml/min. Eluted tetramer fractions were pooled, concentrated and used for activity assay and characterization. All chromatography experiments were carried out using AKTA protein purifier (GE Healthcare, Sweden). SDS polyacrylamide gel electrophoresis was performed according to method of Laemmli on a slab gel containing 12% running gel and 5% stacking gel (Laemmli, 1970).

### Protein estimation and mass spectrometry

Protein concentration was determined by micro BCA assay kit (Pierce, Germany) using BSA as a standard. For enzyme assay and spectral analysis purified protein concentration was calculated by spectroscopic method using λ 280 nm (1 mg/ml = 0.77 for purified asparaginase). Purified recombinant asparaginase was passed through desalting column to remove salts and other small molecules. Recombinant asparaginase (10 μg/ml) dissolved in 1% formic acid was used for mass spectroscopy analysis. Mass spectrum was collected using LC MS MS system (Waters, USA).

### Analytical gel filtration chromatography using HPLC system

Purified recombinant asparaginase was loaded on protein Bio-Sep S-2000 column (Phenomenex, Torrence, USA) attached to HPLC system (Shimadzu, Japan) to analyze oligomeric form of the recombinant protein. For this, a Bio-Sep S-2000 column was equilibrated with 50 mM Tris-HCl, 10 mM NaCl, pH 7.5 and 20 μl of 1.5 mg/ml purified recombinant asparaginase solution was injected in the column. Equilibration and elution was carried out at a flow rate of 1 ml/min. Gel filtration marker proteins (GE Healthcare, Sweden) were applied on the column for estimation of molecular mass of recombinant L-asparaginase.

### Asparaginase activity assay

Asparaginase activity was assayed using method described by Wriston (1985). Briefly, reaction mixture consisted of 50 mM Tris-HCl (pH 8.6) and 8.6 mM L-asparagine incubated at 37°C for 10 min. L-asparaginase enzyme solution (10 μg/ml) was added in reaction mixture and incubated at 37°C. Reaction was stopped by adding 1.5 M trichloro acetic acid at different time points and samples were centrifuged and used for estimation of released ammonia by Nessler's reagent using ammonium sulfate as standard. Time dependent release of ammonia was determined by taking OD measurements at 432 nm and linear range of ammonia release was found to be up to 30 min. An international unit (IU) of L-asparaginase is defined as the amount of enzyme required to release one micromole of ammonia per min under the condition of the assay at saturating substrate concentration. Assays were run in triplicate.

### Fluorescence and Far-UV CD spectroscopy of purified asparaginase

Fluorescence emission spectra of purified recombinant asparaginase were recorded using the Cary Eclipse spectrophotometer (Varian, Australia) attached with Peltier temperature controller. Spectra were recorded on 1 cm path length cuvettes with excitation and emission slit width 5 nm. 25 μg/ml concentrations were used for acquisition of fluorescence emission spectra. Samples were excited at 280 nm and emission spectra were collected from 290 to 400 nm. Far-UV CD spectra were recorded using Jasco-spectropolarimeter equipped with a Peltier temperature controller. Spectra were acquired with a bandwidth of 1 nm, a step size of 1 nm, and an accumulation of 100 nm per min for three scans. Protein concentration used for CD spectroscopy was 0.4 mg/ml. Asparaginase was denatured in different concentrations of GdmCl. Melting curves were recorded at 222 nm during sample heating and consecutive cooling from 20 to 90°C, with 5°C increment in base region and at 1°C in transition region of thermal denaturation.

### Determination of conformational stability of L-asparaginase

Stability of the active asparaginase tetramer was monitored by measurements of its tryptophan fluorescence at various concentrations of denaturants. Purified asparaginase was denatured in different concentrations of urea and GdmCl. In all the denaturation studies, final concentration of asparaginase in denaturing buffer was 10 μg/ml. Fluorescence emission spectra were recorded in a Cary Eclipse spectrophotometer (Varian, Australia). Samples were excited at 280 nm and emission spectra were recorded between 300 and 400 nm. Analysis of denaturation process was carried out by plotting the ratios of fluorescence intensities (319/355 nm) as function of denaturant concentration.

## Results

### Expression of recombinant L-asparaginase in *E. coli*

**Figure 1:**
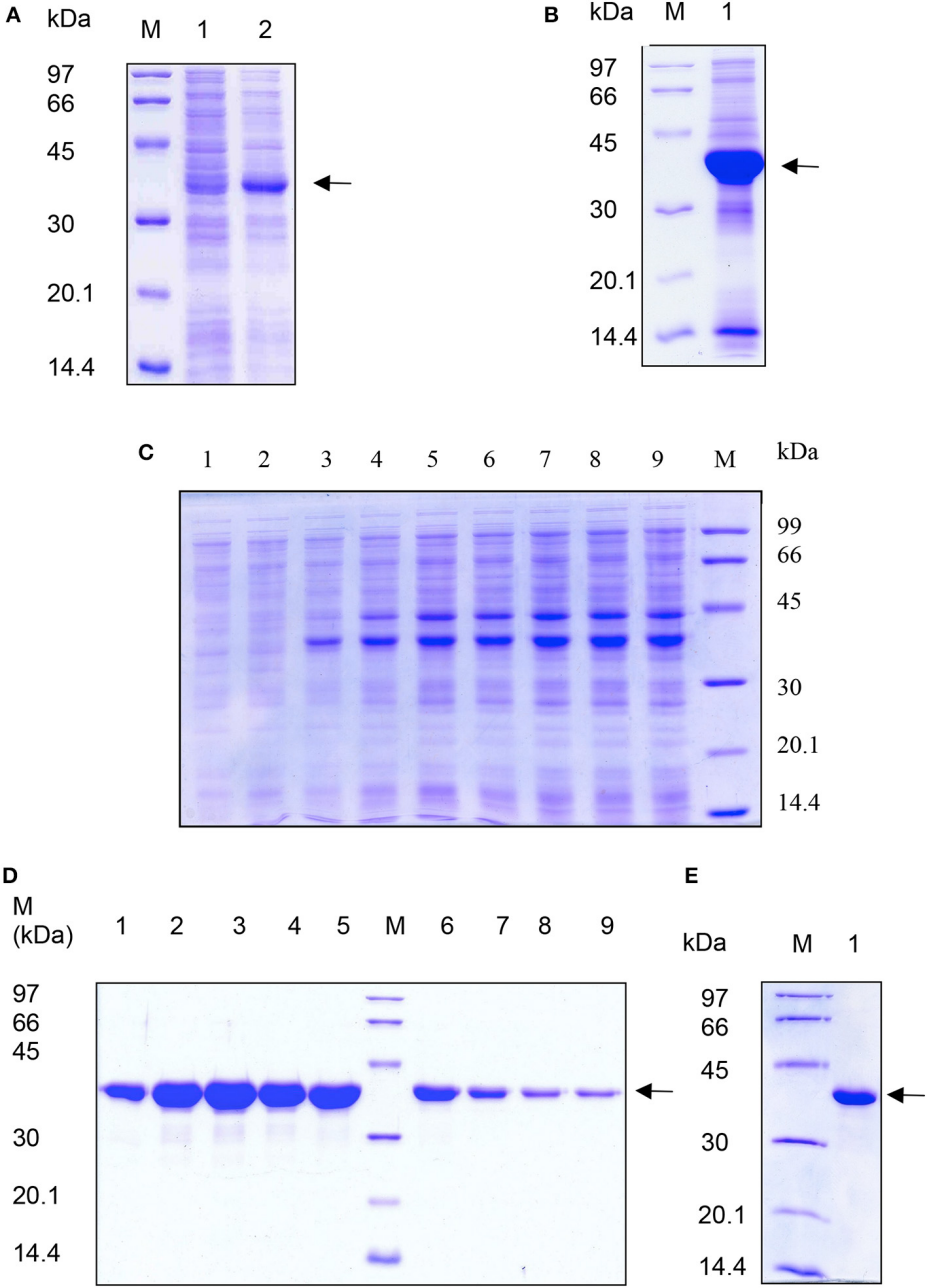
**SDS-PAGE analysis of expression and purification of recombinant L-asparaginase II from *E. coli* IBs**. Samples were analyzed on 12% polyacrylamide gel. Lane M; low molecular weight marker proteins (Pharmacia, Sweden) in **(A–E)**. Arrow indicates recombinant asparaginase. **(A)** Lane 1: uninduced cell lysate; lane 2: induced cell lysate. **(B)** Lane 1: purified IBs. **(C)** Lane 1–9: supernatants of solubilized asparaginase from IBs in 0 to 8 molar urea respectively in 50 mM Tris pH 8.5 buffer. **(D)** Eluted fractions of asparaginase from DEAE ion exchange chromatography. Lanes 1–9 are eluted fractions 1, 3, 5, 7, 9, 11, 13, 15, and 17 from DEAE ion exchange chromatography. **(E)** Lane 1: purified recombinant asparaginase after S-200 gel chromatography.

Transformed *E. coli* cells were induced at optical density of 0.6 at 600 nm with 1 mM IPTG and expression of recombinant asparaginase was checked on 12% SDS polyacrylamide gel. Recombinant L-asparaginase was expressed as a ~37 kDa protein and most of it accumulated as intracellular aggregates (Figure 1A). In shaker- flask culture at OD_600_ of 1.5, around 120 mg of L-asparaginase was produced as IB aggregates. We lysed the cells by sonication in the presence of lysozyme and IB pellet was extensively washed with 0.1% Na deoxycholate. Use of lysozyme during cell lysis helped in better recovery of IB aggregates from cells. Washing the IB pellet with 0.1% deoxycholic acid helped in the removal of contaminating membrane proteins. Purified IB pellet was observed to consist of two major bands on SDS-PAGE gel, one corresponds to recombinant L-asparaginase (85%) and another one to the lysozyme used during IBs isolation (Figure 1B). It was observed that small fraction of lysozyme may stick to IBs during isolation step. This was also observed by others in their study (Peternel and Komel, 2010).

### Refolding and purification of asparaginase from IBs

**Figure 2:**
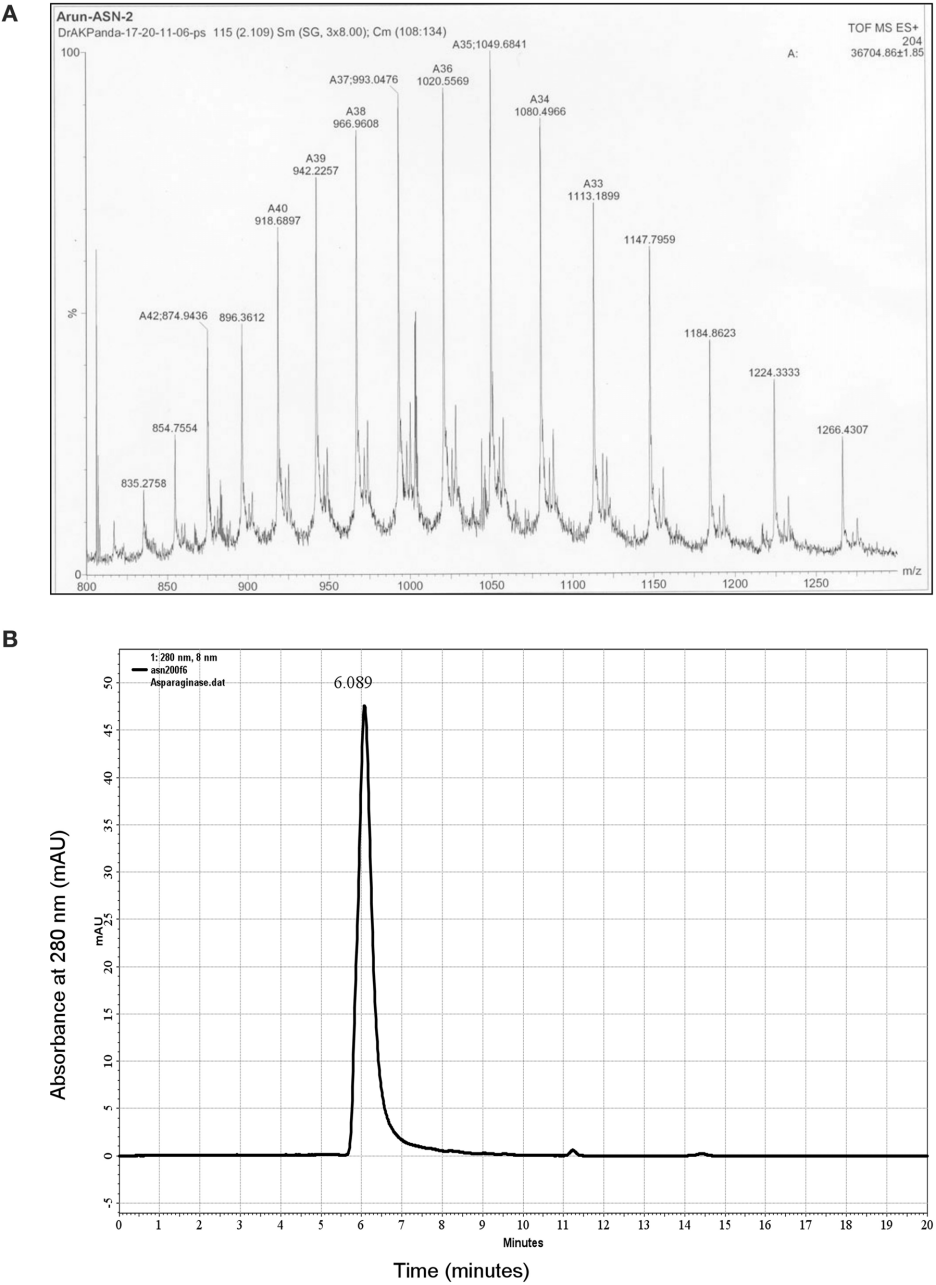
**Characterization of purified asparaginase. (A)** Mass spectrogram of purified asparaginase. Protein was dissolved in 0.1% formic acid and spectrum was obtained from LCMS instrument, Waters, USA. **(B)** HPLC size exclusion chromatogram of purified recombinant asparaginase loaded on Bio-Sep S-2000 column (Phenomenex, USA). Elution profile shows the presence of single peak corresponding to the asparaginase tetramer of ~150 kDa and retention time 6.08 min.

Isolated and purified L-asparaginase IBs were solubilized in different concentrations of urea in 50 mM Tris-HCl buffer at pH 8.5. 4 M urea completely solubilized the recombinant L-asparaginase IBs (Figure 1C). An extra major protein band was observed above the recombinant L-asparaginase band in Figure 1C. This protein band probably represents the host chaperone protein (DnaJ ~40 kDa) expressed during recombinant expression of L-asparaginase. The solubility of asparaginase IBs in 4 M urea containing Tris-HCl was found to be ~10 mg/ml. Solubilized recombinant asparaginase was refolded by pulsatile dilution method. Presence of 0.1 M arginine in refolding buffer reduced protein aggregation, promoted tetramer formation and improved the protein recovery during refolding. Refolded asparaginase was dialyzed and loaded on DEAE ion-exchange chromatographic column. Refolded asparaginase was eluted at a conductance of 8–20 mS/cm. Eluates from ion exchange chromatography were analyzed by SDS-PAGE and found to be 95% pure (Figure 1D). Ion exchange fractions containing asparaginase were pooled and concentrated by ultra-filtration (10 kDa molecular weight cut-off) unit and loaded on S-200 column to separate the monomer and other higher soluble aggregates species from tetramer. The eluted asparaginase was pure (Figure 1E). Maximum step recovery of protein was achieved during solubilization and refolding steps (Table 1). Approximately 60 mg of purely refolded L-asparaginase was recovered from 1 L shaker -flask culture. The overall recovery of the bioactive enzyme from the IBs was ~50%. Mass spectroscopy data analysis showed the molecular weight of purified asparaginase single subunit to be equal to ~36.7 kDa that is equal to calculated molecular weight of recombinant asparaginase with six histidine tags (Figure 2A). To check whether purified recombinant asparaginase consists of a mixture of large molecular aggregates and monomer form other than the native tetramer, purified protein was loaded on BioSep S-2000 column. Purified recombinant asparaginase was eluted at retention time 6.08 min at 1 ml/min flow rate with 100% peak intensity (Figure 2B). Molecular weight of eluted asparaginase was calculated using standard calibration curve plotted using different marker proteins and found to be close to 150 kDa. Molecular weight of purified asparaginase was found to be 8–10 kDa more due to the addition ~2 kDa N-terminal His-tag in each subunit of active tetramer. This result showed that the purified recombinant asparaginase was in tetrameric form.

**Table 1:** **Purification of recombinant *E. coli* L-asparaginase II from IBs (culture volume 1 liter)**.

**Fractions**	**Total protein (mg)**	**Specific activity (IU/mg)^a^**	**Fold purification^b^**	**% Yield**
Total cell protein	580	–	–	–
IBs	118	–	–	100
Solubilized protein	110	–	–	93.2
Refolded protein	97	125	1	82.2
DEAE ion-exchange chromatography	78	160	1.3	66.1
Gel filtration chromatography	60	190	1.5	50.8

a One international unit is defined as amount of enzyme required to produce one micromole of ammonia per min under standard condition of reaction.

b Fold purification was calculated after refolding as the ratio of specific activity at given step of purification to specific activity of refolded sample.

### Enzyme activity and determination of kinetic parameters of purified asparaginase

**Figure 3:**
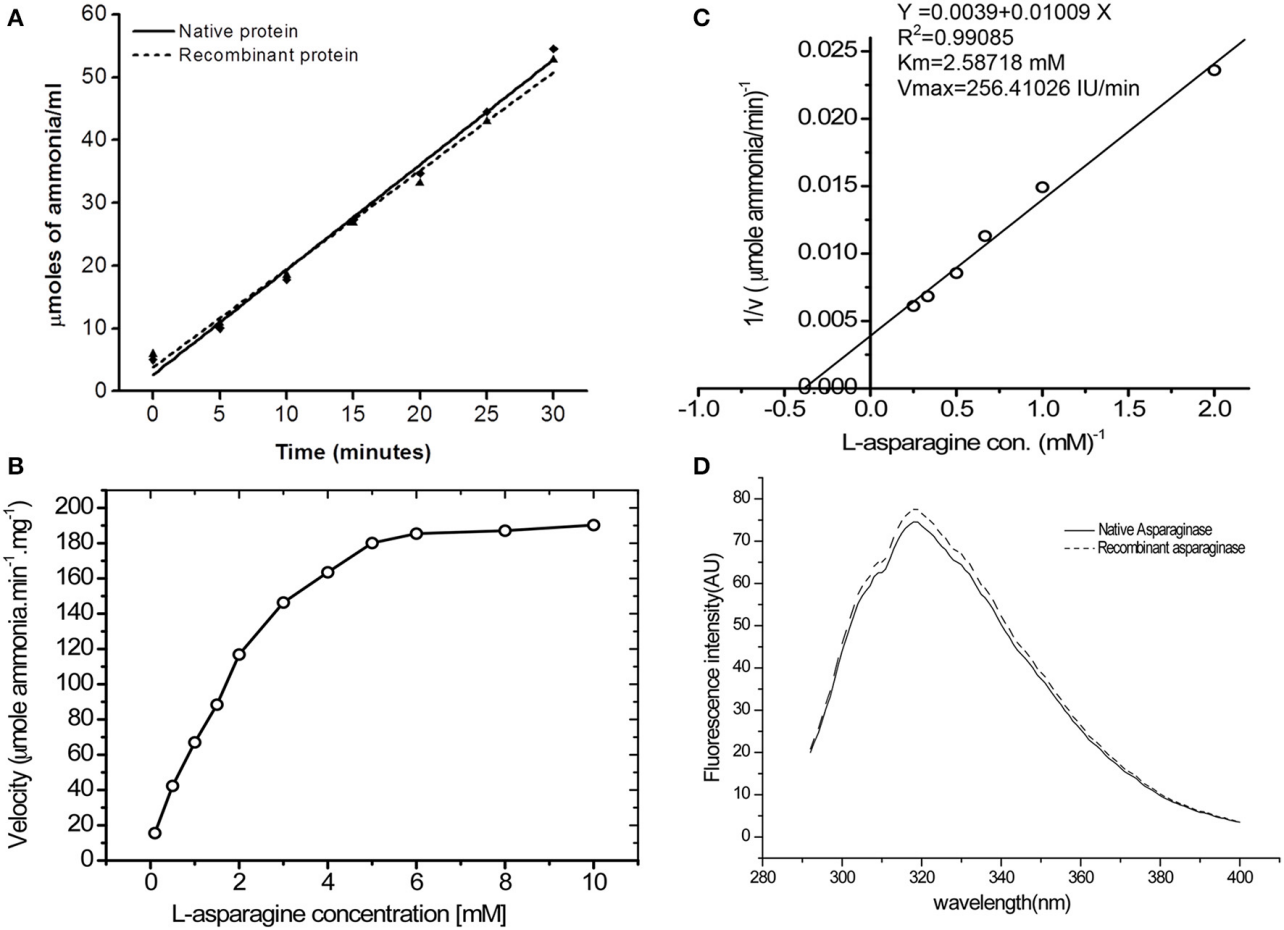
**Bioactivity of purified asparaginase. (A)** Time dependent ammonia release per ml of reaction mixture (Y axis) for native (-♦-) and recombinant (-▴-) asparaginase was calculated by Nessler's reagent method. Amount of asparaginase enzyme in final reaction buffer was 10 μg/ml. **(B)** Effect of L-asparagine concentration on specific activity of recombinant asparaginase. **(C)** Double reciprocal plot between velocity and L-asparagine concentration for determination of Km and Vmax values. **(D)** Fluorescence emission spectra of the native (—) and purified recombinant (−−−−) L-asparaginase II at 15 μg/ml concentration in 50 mM Tris-HCl buffer, pH 7.5. Emission spectra show maxima at 320 nm with two shoulders at 307 and 325 nm at 280 nm excitation wavelengths.

Steady-state kinetic analysis of recombinant L-asparaginase was carried out at saturating substrate concentration of L-asparagine to determine its specific activity. Time duration of the reaction was determined by calculating linear range of ammonia release from both recombinant and native asparaginase (Figure 3A). It was observed that up to 30 min, the amount of ammonia release was linear (*R*^2^ value for native protein on linear fit was 0.991 and for recombinant asparaginase it was 0.990). As the protein was refolded and purified, the specific activity increased from 125 to 190 IU/mg (Table 1). Specific activity of final purified protein was found close to native *E. coli* asparaginase (200 IU) and was around 190 ± 5 IU. The kinetic parameters for purified asparaginase were calculated by determining the specific activity of asparaginase at different concentrations of L-asparagine (0.1–10 mM) (Figure 3B). The Km and Vmax value were calculated from the linear fit of double reciprocal Lineweaver-Burk plot (Figure 3C). The Km and Vmax values for purified protein were found to be 2.58 mM L-asparagine and 256 IU/mg respectively. The fluorescence spectrum of the refolded asparaginase was comparable to that of the native enzyme (Figure 3D). The similarity of specific activities and fluorescence spectrum of the refolded enzyme with the native asparaginase indicated that the recombinant protein has been refolded into native-like conformation.

### Chemical denaturation of asparaginase by urea and GdmCl

**Figure 4:**
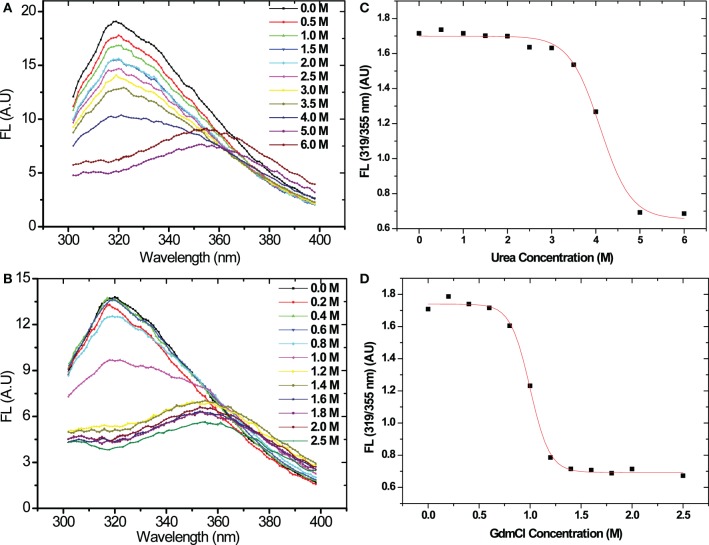
**Fluorescence emission spectra of L-asparaginase denatured in different concentrations of denaturants in pH 8.5, Tris-HCl buffer. (A)** Emission spectra in presence of urea. **(B)** Emission spectra in presence of GdmCl. Colored bars indicate the molar concentrations of denaturants. Λex = 280 nm. Denaturation profile of recombinant asparaginase in **(C)** urea and **(D)** GdmCl.

**Figure 5:**
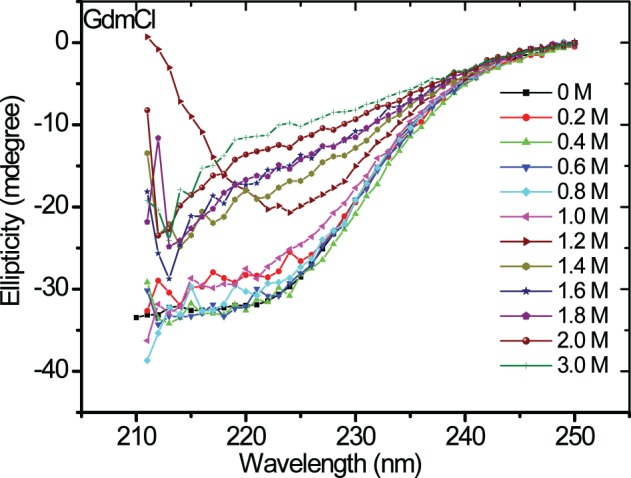
**Far-UV CD spectra of asparaginase in presence of different concentrations of GdmCl**. Colored bars show the concentrations of GdmCl.

Chemical denaturation of asparaginase was probed by fluorescence spectroscopy in the presence of different concentrations of urea and GdmCl. The protein was excited at 280 nm, and emission spectra were recorded between 300 and 400 nm. Fluorescence spectra of asparaginase in the presence of different concentrations of urea and GdmCl are presented in Figures 4A,B. Native asparaginase showed emission maximum at 319 nm. In the presence of 6 M urea or 2 M GdmCl, complete denaturation of the protein was observed with emission maximum at 356 nm that is a characteristic feature of solvent- exposed tryptophan residues in polar environment. Fluorescence intensity was reduced with the increase in urea concentration (up to 4 M) without any significant shift in emission maxima. However, there was a 36 nm red shift in emission maximum of asparaginase at higher than 4 M urea concentrations. Similar denaturation behavior was observed in the presence of GdmCl. However, being a stronger denaturant, loss of fluorescence intensity and red shift in emission maximum occurred at 1.2 M concentration of GdmCl. As asparaginase has characteristic emission maxima wavelength in native and denatured states, denaturation curves of asparaginase were analyzed as ratios of fluorescence intensities at 319/356 nm at different concentrations of urea and GdmCl. Asparaginase showed cooperative denaturation profile in urea and GdmCl (Figures 4C,D). It can be concluded that asparaginase is more susceptible to GdmCl denaturation in comparison to urea. This conclusion was further supported by the denaturation profiles of asparaginase in presence of GdmCl observed with circular dichroism (CD) spectroscopy (Figure 5).

**Figure 6:**
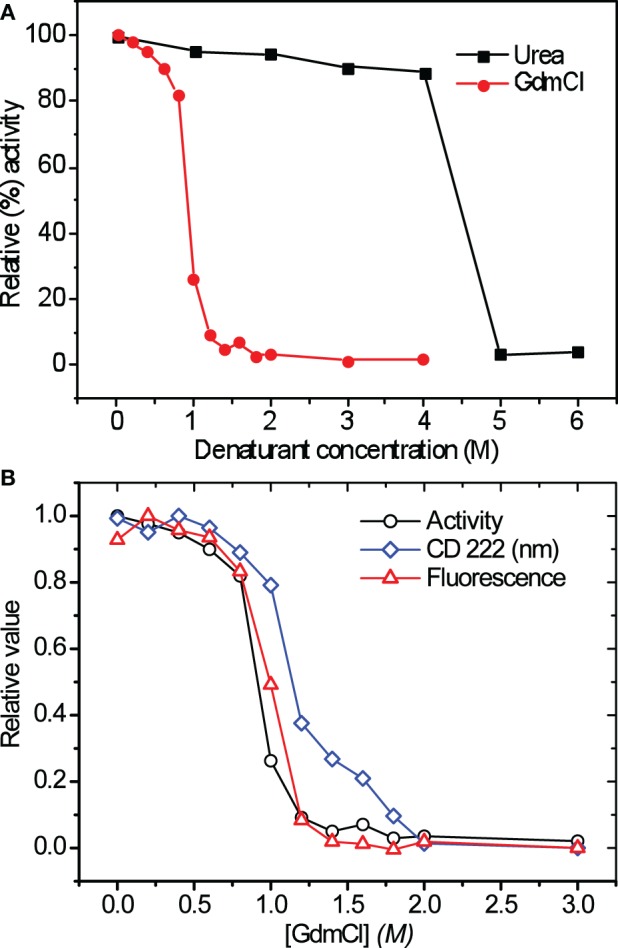
**(A)** Relative activity of asparaginase in presence of different concentrations of urea and GdmCl (activity in absence of denaturants was considered to be 100%). The colored bars indicate the denaturants. **(B)** GdmCl induced denaturation curves of recombinant asparaginase. The appropriate fraction of native protein (relative values) was plotted vs. GdmCl concentration.

We determined the asparaginase enzymatic activity in the presence of denaturants such as urea and GdmCl at different concentrations to gain information on the stability of quaternary structure. The denaturant concentration-dependent activity profile of asparaginase is shown in Figure 6A. No significant loss in activity of asparaginase till 4 M urea was observed. Similarly, asparaginase retained 80% activity in 0.8 M GdmCl. The GdmCl concentration-dependence of fluorescence spectra, CD signal at 222 nm and enzyme activity of asparaginase was compared (Figure 6B). Fluorescence signal and activity were found to be in agreement. However, the CD signal at 222 nm showed a biphasic denaturation behavior in which first transition state was similar to the fluorescence and activity profile. The second transition state was observed between 1.4 and 1.8 M concentrations of GdmCl where the protein retained some proportion of secondary structures.

### Thermal denaturation and determination of melting temperature

**Figure 7:**
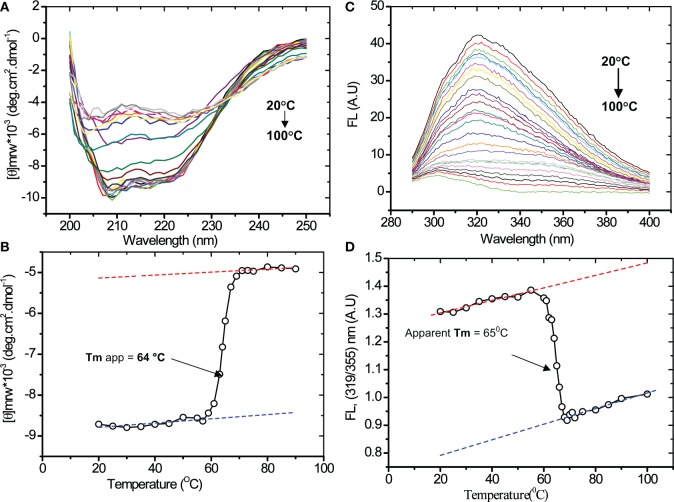
**Thermal denaturation of recombinant asparaginase. (A)** Far-UV CD spectra of asparaginase at different temperatures at pH 8.0. **(B)** Thermal denaturation curve of asparaginase plotted between CD 222 nm signal and temperature. **(C)** Fluorescence spectra of asparaginase at different temperatures at pH 8.0. **(D)** Thermal denaturation curve of asparaginase was plotted between FL (319/355) nm and temperature. Tm values are indicated by arrow.

Thermal stability of purified recombinant asparaginase was probed by CD and fluorescence spectroscopy. The CD spectra of asparaginase were recorded at different temperatures (Figure 7A). Samples were left for 5 min for equilibration at each point, and three scans were recorded. In pre-and post-transition range of thermal denaturation, scans were performed at 5°C intervals and 1°C intervals within the transition range. CD 222 nm signals were plotted against temperature (Figure 7B). Thermal denaturation curves showed the loss of CD signal at start of 57°C and gradually decreased to the lowest value at 70°C. Further increase in temperature beyond 70°C had no significant effect on CD 222 nm signal. The melting temperature (Tm) of asparaginase was found to be 64°C at pH 8.0. Similarly, the thermal denaturation of asparaginase was probed by acquiring the fluorescence spectra at different temperatures (Figure 7C). Ratio of fluorescence intensities at 319 and 355 nm (FL at 319/355 nm), was plotted against temperature (Figure 7D). The denaturation curve showed the increase in the FL at 319/355 nm) value up to 57°C. After that, there was a sharp decrease in this value. In post- transition range, a similar trend was observed as in pre-transition range. The curve fitting showed that the apparent Tm to be 65°C. It was close to the Tm value calculated from thermal denaturation curve plotted from CD signals.

## Discussion

Refolding yield of bioactive proteins from IBs of *E. coli* are in general very low and contribute toward a major cost for production of recombinant proteins (Datar et al., 1993). However, expression of protein as IBs has the advantages of very high level of protein expression and resistance to protease attack. Thus, IB expression helps in purifying the denatured protein with a lower number of operating steps. If suitable high throughput refolding procedure can be developed for a particular protein, IB formation will be very helpful in the production of recombinant protein. Such an approach will be more beneficial for oligomeric proteins where the refolding yields are even lower. Strategy used to recover bioactive protein from IBs mainly involves four steps: isolation of IBs from *E. coli* cells; solubilization of the IB aggregates; refolding and purification of the solubilized protein. Among these steps, solubilization of the protein aggregates and refolding of the solubilized protein are the most crucial steps and need careful attention for high recovery of protein. Aggregation leading to low recovery of the recombinant protein occurs due to the use of sub-optimal refolding procedure. It is expected that mild solubilization of the IB aggregates using low concentration of denaturants followed by pulsatile refolding of the solubilized protein would be an ideal approach for maximal recovery of bioactive proteins from the IBs.

In this study, *E. coli* L-asparaginase II was expressed as IB aggregates in *E. coli*, purified by detergent washing and solubilized using only 4 molar urea solution. Maximum of 10–11 mg/ml of the protein could be solubilized at 4 M urea solution. The solubilized protein was subsequently refolded into native tetrameric form using pulsatile dilution method. Around 50% of IB protein could be refolded into bioactive tetrameric protein using the mild solubilization procedure. The production data are comparable to that reported for soluble expression using LB medium in shaker-flask culture (Khushoo et al., 2004). It is expected that if such expression system are optimized using high cell density fermentation followed by the present IB refolding process, the volumetric productivities will be even better. Crystal structure of *E. coli* asparaginase shows that the active site is present between subunits of intimate dimer formed by interaction of N-terminal domain of one subunit with C-terminal domain of the other subunit. Each dimer has two active sites, but only tetramer shows the activity. Apart from this, 0.1 M arginine in refolding buffer also promoted the formation of tetramer and reduced the extent of protein aggregation. It showed that the arginine not only helped in preventing aggregation during refolding but also promoted the formation of correctly folded subunits for proper association into tetrameric form. Fluorescence spectrum of the refolded asparaginase was similar to that of native asparaginase indicating the formation of proper quaternary structure of the recombinant asparaginase after refolding.

According to the crystal structure of asparaginase, α-helices are predominantly present on the surface of tetramer. Active site of asparaginase is present on the interface of subunits with tryptophan residues at the core of N-terminal domains of monomers near the active site. The cooperative fluorescence and activity profile and biphasic nature of CD spectra in the presence of different concentrations of GdmCl could be possible if, during the denaturation, dissociation of subunits precedes the complete loss of secondary structures into monomers. Protecting the existing secondary structure element of the protein during solubilization thus favors the association monomer into oligomeric form and results in the formation of tetrameric bioactive protein.

## Conclusions

Understanding the solubilization profile of IB aggregates at different denaturation concentration helped in designing a mild solubilization procedure. Such a mild process protected the existing native-like protein structure and thus helped in improved recovery of bioactive protein during refolding. Solubilized asparaginase from the IBs was refolded into tetrameric form having high specific enzymatic activity by optimization of solubilization and refolding conditions. Purified asparaginase was found to be active. Kinetic parameters of the enzyme were determined. Purified asparaginase was characterized for its denaturation profiles in the presence of urea and GdmCl. Thermal denaturation of the purified asparaginase was also studied, and the Tm was determined to be about 64°C. Mild solubilization of IB protein is thus the key requirements in high throughput recovery of IB proteins into bioactive form. This concept thus can be used for recovery of bioactive oligomeric proteins from the IBs of *E. coli.*

### Conflict of interest statement

The authors declare that the research was conducted in the absence of any commercial or financial relationships that could be construed as a potential conflict of interest.
